# COVID-19 cleaning protocol changes, experiences, and respiratory symptom prevalence among cleaning services personnel

**DOI:** 10.3389/fpubh.2023.1181047

**Published:** 2023-09-14

**Authors:** Amanda M. Wilson, Yoonhee Jung, Sydney A. Mooneyham, Ivana Klymko, Josie Eck, Carlos Romo, Vineeth R. Vaidyula, Sam J. Sneed, Lynn B. Gerald, Paloma I. Beamer

**Affiliations:** ^1^Department of Community, Environment and Policy, Mel and Enid Zuckerman College of Public Health, University of Arizona, Tucson, AZ, United States; ^2^Southwest Environmental Health Sciences Center, University of Arizona, Tucson, AZ, United States; ^3^Department of Health Promotion Sciences, Mel and Enid Zuckerman College of Public Health, University of Arizona, Tucson, AZ, United States; ^4^Honors College, Virginia Commonwealth University, Richmond, VA, United States; ^5^Population Health Sciences Program, Office of the Vice Chancellor for Health Affairs, University of Illinois Chicago, Chicago, IL, United States; ^6^Breathe Chicago Center, University of Illinois Chicago, Chicago, IL, United States

**Keywords:** janitor, custodian, maid, disinfection, respiratory disease, pandemic

## Abstract

**Introduction:**

Cleaning protocols were changed in response to the COVID-19 pandemic with unknown occupational health impacts. There is evidence that COVID-19 transmission risks from contaminated surfaces are low and that exposure to cleaning products can increase risks of work-related asthma. The study objective was to investigate relationships between reported COVID-19-related changes in cleaning protocols and prevalence of asthma-related respiratory symptoms for asthmatic and non-asthmatic janitors and maids. A secondary objective was to characterize experiences of respiratory symptoms associated with cleaning and barriers to personal protective equipment (PPE) use.

**Methods:**

Employees from two Tucson-based maid service companies (approximately 30 personnel in total) and one Phoenix-based school district (>300 janitors/custodians) were invited to participate in a written survey and/or a one-on-one interview in Spanish or English. Fisher’s exact tests (*α* = 0.05) were used to test for statistically significant associations between reported respiratory symptoms by self-reported physician-diagnosed asthma status and changes in cleaning protocols. Interviews were transcribed and then analyzed by at least two researchers in English or Spanish.

**Results:**

Eighty-three percent reported that cleaning protocols had changed during COVID-19, with the two most reported changes including increased cleaning frequency (92%) and change of application type (e.g., fog, spray, wipe) (53%). There was a statistically significant association between multiple respiratory symptoms and self-reported physician diagnosed asthma. Reporting a type of application change (e.g., fog, spray, wipe) and being awakened during the night by attack/episode of cough were statistically significantly associated (*p* = 0.04). Interviews elucidated respiratory issues related to fogging devices.

**Discussion:**

This study provides preliminary evidence that changes in cleaning and disinfection protocols during COVID-19 (namely, the use of fogging/mechanical spraying devices) may have had negative impacts on the health of workers in the cleaning industry with little benefit to reducing COVID-19 risks. Further research is needed to evaluate the generalizability of our findings across larger geographical areas and to develop guidance for employers and employees on how to protect and promote respiratory health.

## Introduction

There is evidence that exposure to cleaning and disinfection products can increase risks of work-related asthma outcomes. Much of this evidence is for health care environments (1–4), including higher odds of poor asthma control for nurses who use disinfectants to clean medical instruments (3), and increased odds of physician-diagnosed asthma for those exposed to quaternary ammonium compounds (5). There is also evidence of higher rates of asthma among those in cleaning industries relative to other industries (6–8).

Early in the COVID-19 pandemic, it was thought that fomites, or surfaces capable of harboring pathogens, may be a driver of COVID-19 transmission. This led to increased cleaning and disinfection frequency and intensity in many different environments, with increased calls to poison centers related to cleaning and disinfection exposures (9). Within approximately a year of the pandemic, it was demonstrated that fomites likely do not contribute greatly to COVID-19 transmission, especially in comparison to other routes, such as the airborne route (10–12). The Centers for Disease Control and Prevention then released a scientific brief stating that risks from fomites are likely less than 1/10,000 for a single fomite touch, informed by quantitative microbial risk assessments (13–15). Despite this pivot away from fomites, many places continued to implement new cleaning and disinfection protocols in response to COVID-19. The increased cleaning and disinfection, despite low risks from fomites, is an example of “risk–risk tradeoff,” or the increase of one risk and decrease in another because of a behavior change or intervention. In this case, increased cleaning and disinfection increased risk of asthma outcomes while decreasing risk of COVID-19 transmission, even if these risks vary greatly in magnitude.

The perceptions of COVID-19 risk vs. asthma risk related to cleaning and disinfection are relatively unknown. Differences in asthma prevalence among cleaning services personnel in a variety of environments are also unknown, where previous research has traditionally grouped cleaning personnel into one occupational category. Since different environments have implemented COVID-19 cleaning and disinfection protocols differently, variability in asthma prevalence and perceptions related to health risks from increased or decreased cleaning and disinfection is expected.

Evaluating associations between respiratory symptoms and changes in cleaning and disinfection protocols in response to the COVID-19 pandemic will help inform emergency preparedness for future pandemics. These insights may also highlight the need for further education on how to properly clean/disinfect to limit exposure to chemicals that can increase risks of work-related asthma. Additionally, addressing potential barriers to proper use of PPE or other interventions to reduce exposures while cleaning and disinfecting is vital for improving the safe and effective implementation of new cleaning and disinfection protocols in response to future outbreaks or pandemics.

### Study Objective

The objectives of this study were to (1) measure asthma prevalence among cleaning services personnel, and (2) characterize changes in cleaning and disinfection protocols in a variety of environments during COVID-19, using a mixed methods approach in which interviews were used to contextualize survey results.

## Methods

### Recruitment

Businesses and school districts were approached with the opportunity to allow their employees to participate in the survey and interview research opportunities (described in detail below). Two Tucson-based maid service companies (with approximately 30 personnel in total) and one Phoenix-based school district (with over 300 janitors/custodians) provided written support of the project. The University of Arizona and school district Institutional Review Boards approved the research protocol (protocol #: STUDY00000690). Participants were eligible to participate in a survey or interview if they were 18 years or older and had been working in a cleaning role for at least 1 year, regardless of asthma status. Participants had the option of participating in one or both opportunities and were compensated for each separately. Eligibility was confirmed at the beginning of the survey and interview. If someone indicated they did not meet eligibility criteria, their survey data were not included in the study and/or the interview did not proceed. A convenience sample was used due to challenges in accessing this vulnerable population, where a high proportion of eligible individuals were anticipated to be immigrants and with low access to technology.

One hundred fifty surveys were hand delivered to participating organizations (30 to the maid companies, combined, over 120 to the school district) with pre-paid postage on envelopes for participants to mail back completed surveys. Businesses and the school district were offered more packets if needed. These packets also included information on how to contact research personnel to schedule an interview. Consent forms and directions on completing the survey were included in English and Spanish, along with contact information for expressing interest in participating in an interview. Participants were compensated with gift cards for mailing back completed surveys (with no identifying information other than their email address to which to send compensation) and/or completing an interview.

### Survey and interview question design

Surveys were developed in English and then translated to Spanish, with at least 2 Spanish-speaking individuals reviewing the translation. Survey questions included a validated 8-item predictor of asthma (16), and additional questions were included from the previously validated survey, also regarding asthma-related symptoms (16). The survey did not include questions about the timing of asthma diagnosis. Questions about changes in symptoms or cleaning/disinfection protocols due to COVID-19 and participant demographics (gender, race, ethnicity, age) were included. Interview questions were consistent with survey questions in inquiring about asthma status (but not timing of diagnosis, although some participants disclosed this independently), respiratory symptoms and changes to cleaning/disinfection protocols during COVID-19, along with questions regarding personal protective equipment (PPE) use, management of respiratory symptoms at work, and risk perceptions of asthma from increased cleaning/disinfection and/or risk of infection from unclean surfaces. Questions were also included about cleaning/disinfection practices in the home environment and changes due to COVID-19.

### Survey analysis

Descriptive statistics were calculated for demographic variables, work environment, and cleaning/disinfection protocol questions (e.g., number of hours spent cleaning pre- and during the COVID-19 pandemic). Fisher’s exact tests (*α* = 0.05) were used to test for statistically significant associations between proportions of those who reported experiencing respiratory symptoms by (1) physician-diagnosed asthma status (yes or no), and whether they reported, (2) a change in cleaning protocols during COVID-19 (yes or no), (3) increased cleaning frequency (yes or no), (4) change in cleaning/disinfection product (yes or no), or (5) change in application (i.e., spray, wipe, fog) (yes or no). Those who chose “I do not know” or “prefer not to respond” to any of the previously listed variables were not included in this portion of the analysis, since the focus was on associations of asthma and reported changes in cleaning and disinfection protocols with respiratory symptoms as opposed to associations with uncertainty regarding cleaning and disinfection protocol changes or preferences not to disclose.

### Interview analysis

Interview recorded audio was transcribed by research personnel who were native and/ or fluent and trained speakers in the language in which the interview was conducted. Transcripts were then analyzed separately by two researchers for the following content:

- Participant demographics (gender, age, ethnicity, and race).
- Self-reported asthma diagnosis.
- Type of work environment, type of cleaning conducted at work (handheld spray, wipes, fogging or motorized sprayers, other), and surfaces cleaned at work.
- Type of cleaning conducted at home (sprays, wipes, other) and surfaces cleaned at home.
- Changes in cleaning/disinfection at work due to COVID-19 (change in frequency, personal protective equipment (PPE) changes, change in product, change in application type (e.g., use of motorized sprayer/fogger), change in types of surfaces, heightened awareness, other, none).
- Challenges/strategies related to PPE use or respiratory symptoms at work and at home.
- Concerns/issues with inhalation of cleaning/disinfection chemicals at work and at home.
- Other symptoms or concerns from cleaning/disinfection at work and at home.
- Concerns/issues with COVID-19.
- Thoughts regarding risk–risk tradeoffs associated with cleaning and disinfection.
- Awareness of asthma as a risk from cleaning and disinfection activities.

These content categories were determined using an inductive approach, since little research has been done on this topic for informing a deductive approach. More details on how responses were categorized can be found in Supplementary Table S1. Spanish transcripts were analyzed in Spanish to reduce loss of context or meaning that could occur through translation. Consistency in categorization of responses by the two researchers were compared, and consensus was reached through discussion. Key quotes that supported the categorization of content topics were identified and agreed upon by at least two research personnel and translated to English for the manuscript.

## Results

### Survey participant demographics

Fifty-nine participants completed the survey out of 150 surveys that were given to businesses, reflecting a 39% response rate. We did not collect information on specific industry (school district vs. maid service company) to protect employers and employees. However, we did collect information on the types of spaces cleaned. The two most reported types of indoor spaces cleaned included bathrooms (96%, 55/57) and classrooms (89%, 51/57). The greatest proportions of survey participants were female (54%, 32/59), age 56–65 years old (27%, 16/59), and non-White or other (did not select a race) Hispanic (54%, 32/59). Twenty-seven percent reported over 15 years in a cleaning industry role, followed by 24% with 6–10 years of experience. Fourteen percent (8/59) of participants reported having physician-diagnosed asthma. While larger than the national proportion of adults with asthma (8.4%) (17), this was not a statistically significant difference (X-squared = 1.43, df = 1, value of *p* = 0.23) using a one sample proportions test. Fourteen percent of all participants also reported taking medication for asthma. All survey participant demographics can be seen in Table 1.

**Table 1:** Participant demographics.

Variable	Category	% (n/total)
Survey participants (*n* = 59)		
Gender	Male	44% (26/59)
	Female	54% (32/59)
	Non-binary	0% (0/59)
	Other	0% (0/59)
	Prefer not to respond	2% (1/59)
Age	18–25	12% (7/59)
	26–30	10% (6/59)
	31–35	2% (1/59)
	36–40	3% (2/59)
	41–45	14% (8/59)
	46–50	12% (7/59)
	51–55	14% (8/59)
	56–65	27% (16/59)
	65+	5% (3/59)
	Prefer not to respond	2% (1/59)
Race and ethnicity	Non-Hispanic Black or African American	2% (1/59)
	Hispanic, American Indian or Alaska Native	3% (2/59)
	White, Non-Hispanic	17% (10/59)
	White, Hispanic	20% (12/59)
	Non-white/other (did not specify race) Hispanic	54% (32/59)
	Prefer not to respond	3% (2/59)
	Native Hawaiian or Pacific Islander	0% (0/59)
	Asian	0% (0/59)
Physician-diagnosed asthma	Yes	14% (8/59)
	No	80% (47/59)
	I do not know/Prefer not to respond	5% (3/59)
Currently taking medication for asthma	Yes	14% (8/58)
	No	86% (50/58)
Frequency of respiratory symptoms (cough, wheezing, chest tightness, shortness of breath) at work during COVID-19	Stayed the same	59% (35/59)
	Decreased	10% (6/59)
	Increased	5% (3/59)
	Prefer not to answer	22% (13/59)
Number of years in a custodial or janitorial role	1–2	22% (13/59)
	3–5	14% (8/59)
	6–10	24% (14/59)
	11–15	10% (6/59)
	>15	27% (16/59)
	Prefer not to answer	2% (1/59)
Types of spaces cleaned*	Patient rooms in healthcare	14% (8/57)
	Offices	81% (46/47)
	Classrooms	89% (51/57)
	Bathrooms	96% (55/57)
	Other	44% (25/57)
Cleaning protocols changed during COVID-19	Yes	83% (48/58)
	No	9% (5/58)
	I do not know	9% (5/58)
Types of protocol changes*	Cleaning/disinfection was more frequent	92% (54/59)
	The types of surfaces cleaned changed	51% (30/59)
	The types of products changed	44% (26/59)
	The type of application changed (fog, spray, wipe)	53% (31/59)
Interview participants (*n* = 11)		
Gender	Male	45% (5/11)
	Female	55% (6/11)
	Non-binary	0% (0/11)
	Other	0% (0/11)
Race and ethnicity	Non-Hispanic Black or African American	0% (0/11)
	Hispanic American Indian or Alaska Native	0% (0/11)
	White, Non-Hispanic	18% (2/11)
	White, Hispanic	18% (2/11)
	Non-white/other (did not specify race) Hispanic	64% (7/11)
	Prefer not to respond	0% (0/11)
	Native Hawaiian or Pacific Islander	0% (0/11)
	Asian	0% (0/11)
Age	18–25	0% (0/11)
	26–30	9% (1/11)
	31–35	0% (0/11)
	36–40	9% (1/11)
	41–45	18% (2/11)
	46–50	36% (4/11)
	51–55	0% (0/11)
	56–65	27% (3/11)
	65+	0% (0/11)
Physician diagnosed asthma	Yes	36% (4/11)
	No	64% (7/11)

*Note that participants can report having cleaned in more than 1 environment or more than 1 change in protocol changes, so the total % will be greater than 100% for these categories.

### Survey results

While 59% of participants reported that the frequency of respiratory symptoms had stayed the same during COVID-19, 5% reported an increase, 10% reported a decrease, and 22% preferred not to respond. Eighty-three percent reported that cleaning protocols had changed during COVID-19, with the two most reported changes including increased cleaning frequency (92%) and change of application type (e.g., fog, spray, wipe) (53%). There were statistically significant relationships between the proportion of those reporting physician-diagnosed asthma and those experiencing trouble with breathing (*p* < 0.01), wheezing/whistling in the chest at any time in the past 12 months (*p* < 0.01), wheezing/whistling in the chest not associated with a cold in the last 12 months (*p* < 0.01), missing days from work due to asthma in the last 12 months (*p* = 0.01), being awakened during the night by attack/episode of chest tightness (*p* = 0.04), having an attack/episode of shortness of breath while at home at any time in the last 12 months (*p* = 0.03), changes to shortness of breath when away from work for any time in the last 12 months (*p* = 0.01), and getting chest tightness when near animals, feathers, or in a dusty part of one’s house (*p* < 0.01) (Table 2). There were no statistically significant relationships between proportions of those reporting respiratory symptoms and reporting cleaning protocol change, increased cleaning frequency, or change in type of product. However, there was a statistically significant association between proportion of those reporting a type of application change (e.g., fog, spray, wipe) and those being awakened during the night by attack/episode of cough (*p* = 0.04), where 82% (14/17) of those who reported a change in application type experienced this symptom vs. only 20% (4/20) of those who did not report a change in application type reporting this symptom (Table 2). Of these 14 individuals, three reported physician diagnosed asthma, while nine reported not having physician diagnosed asthma and two preferred not to respond.

**Table 2:** Respiratory symptom responses for all participants, by physician-diagnosed asthma status, and by reported change in cleaning protocol.

Respiratory symptom question		Physician-diagnosed asthma		*p*-value	Cleaning protocol change		*p*-value	Increased cleaning frequency		*p*-value	Types of cleaning products have changed		*p*-value	Type of application changed (fog, spray, wipe)		*p*-value
		Yes	No		Yes	No		Yes	No		Yes	No		Yes	No	
**Ever had trouble with breathing**	Yes	7	9	**<0.01**	12	2	0.62	17	1	1	11	7	0.09	9	9	1
	No	1	33		31	3		33	3		13	23		18	18	
If yes, trouble was brought on by work environment	Yes	1	2	0.43	2	0	1	3	0	1	0	3	0.26	2	1	0.60
	No	4	23		24	4		27	3		13	17		14	16	
**Wheezing/whistling in chest at any time in the last 12 months**	Yes	7	9	**<0.01**	11	1	1	14	0	0.56	8	6	0.37	9	5	0.35
	No	1	36		33	4		36	4		17	23		18	22	
**Wheezing/whistling in chest when you did not have a cold in the last 12 months**	Yes	4	2	**<0.01**	6	0	1	6	0	1	4	2	0.39	5	1	0.20
	No	3	42		40	5		44	5		21	28		24	25	
**Miss any days due to asthma in the last 12 months**	Yes	3	2	**0.01**	4	0	1	5	0	1	3	2	0.64	3	2	1
	No	4	43		40	5		45	5		21	29		25	25	
Attack/episode of shortness of breath in the last 12 months	Yes	2	6	0.59	7	0	1	8	0	1	6	2	0.12	5	3	0.71
	No	6	37		38	5		43	4		19	28		24	23	
**Awakened during the night by attack/episode of cough**	Yes	4	12	0.07	15	1	1	18	0	0.16	11	7	0.15	14	4	**0.04**
	No	2	33		30	4		32	5		14	23		17	20	
**Awakened during the night by attack/episode of shortness of breath**	Yes	4	5	**<0.01**	8	0	0.57	9	0	0.57	6	3	0.27	5	4	0.73
	No	2	37		34	5		37	5		17	25		20	22	
**Awakened during the night by attack/episode of chest tightness**	Yes	3	4	**0.04**	6	0	1	7	0	1	2	5	0.68	5	2	0.25
	No	3	36		34	5		38	4		19	23		19	23	
**Attack/episode of shortness of breath while at home at any time in last 12 months**	Yes	4	7	**0.03**	8	1	1	12	0	0.57	8	4	0.10	7	5	0.53
	No	3	38		36	4		39	4		15	29		20	23	
Attack/episode of shortness of breath while at work at any time in last 12 months	Yes	3	8	0.15	9	1	1	12	0	0.57	7	5	0.34	9	3	0.10
	No	4	37		36	4		39	5		18	26		19	25	
**While you were away from work at any time in the last 12 months, was your shortness of breath worse, better, or unchanged?**	Worse	0	0	**0.01**	0	0	0.53	0	0	0.52	0	0	0.66	0	0	1
	Better	4	2		5	1		5	1		2	4		3	3	
	Unchanged	4	27		27	3		28	3		16	15		17	14	
When you are near animals (cats/dogs/horses), feathers (pillows/quilts/duvets), or in a dusty part of your house, do you ever get itchy eyes?	Yes	6	15	0.06	20	1	0.37	21	2	1	12	11	0.28	16	7	0.09
	No	2	26		23	4		27	3		11	19		13	17	
**When you are near animals (cats/dogs/horses), feathers (pillows/quilts/duvets), or in a dusty part of your house, do you ever get tightness in your chest?**	Yes	4	1	**<0.01**	4	0	1	5	0	1	3	2	0.64	3	2	1
	No	2	40		37	5		40	5		18	27		22	23	
When you are near trees, grass, or flowers, or when there is a lot of pollen around, do you ever get itchy or watery eyes?	Yes	5	18	0.45	23	2	0.67	24	2	1	14	12	0.17	16	10	0.27
	No	3	23		21	3		25	2		9	18		12	15	
Number of years in custodial/janitorial role	1–2	1	12	0.51	9	1	0.13	11	2	0.82	5	8	0.56	6	7	0.22
	3–5	1	6		8	0		8	0		5	3		7	1	
	6–10	1	12		14	0		13	1		4	10		8	6	
	11–15	2	4		5	0		6	0		2	4		3	3	
	15+	3	11		11	4		14	2		8	8		6	10	
Change in frequency of symptoms during COVID-19	Stayed the same	4	28	0.34	29	3	0.56	34	1	0.37	15	20	0.75	18	17	0.74
	Decreased	1	4		3	1		5	1		3	3		2	4	
	Increased	1	2		3	0		3	0		2	1		2	1	

Bolded categories and *p* values indicate statistical significance (*α* = 0.05).

### Interview participant demographics

Eleven participants completed an interview out of 150 survey packets that included information on how to participate in an interview, reflecting a 7% response rate. Information on specific industry (maid vs. school janitor/custodian) was not collected to protect employers and employees. However, types of environments in which participants conducted cleaning included nurse’s offices in schools, exam areas, bathrooms, offices, classrooms, hallways, residential homes, school kitchens, and gymnasiums. Fifty-five percent were female, and 45% were male. Most participants (64%, 7/11) were non-White/other (race not specified) Hispanic, with 18% (2/11) being White Hispanic, and 18% (2/11) being White non-Hispanic (Table 1). The largest proportion of participants were 46–50 years old (36%, 4/11), followed by 56–65 years old (27%, 3/11). Four out of eleven reported having been diagnosed with asthma, while seven did not. Of those diagnosed, two were diagnosed as children and two were diagnosed as adults.

### Interview results

Four participants noted the use of fogging/motorized sprayer cleaners. Nine reported changes in cleaning frequency, with a participant stating, *“Just a lot more disinfecting everywhere, especially obviously on- on high touch areas.”* Eight reported changes in personal protective equipment (PPE) protocols, with a participant stating, *“They’re more on you,”* regarding supervisors enforcing compliance with glove use. Other reported changes included change in application type (27%, 3/11), change in type of surfaces cleaned (27%, 3/11), and heightened awareness of the need for improved hygiene to avoid COVID-19 transmission (64%. 7/11), with a participant stating, *“Siempre habíamos limpiado; ahora se limpia más a profundo”* (English translation: We had always cleaned; now we clean more in depth).

Six of the 11 participants reported concerns or issues with inhalation of cleaning and disinfection chemicals at work, with participants stating:

*“I noticed, after we were doing the-the COVID, especially when we- we would fog a classroom, the aerosol would kind of get in my chest and kind of give me a tight chest. And, you know, almost kind of a mild asthma attack.”*

*“Tenemos que traer nuestra mascarilla porque al momento de no usarlo sí se siente síntomas, así como que, como que te cala eso en el pecho.” (English translation: “We have to bring our mask because when we do not use it, we do feel symptoms, like, it kind of seeps into your chest”).*

One participant also noted an issue with skin irritation from the fogging/mechanical spraying devices and using garbage bags to cover their arms:

*“I put, um, the garbage bags, and made a little hole, put my hand through that, and put the gloves around it so it would cover my arms more and all that. But even with that, just because it’s a fine mist, you know, no matter how- how good you do, it’s in the air,* versus*- So it’s going to be around you, and so we all got a little itchy at times.”*

When discussing challenges or strategies related to PPE and/or respiratory symptoms at work, some participants noted difficulty breathing with masks:

*“I mean, we are moving furniture. We’re picking up garbage anywhere from, you know, really lightweight to, you know, 25-, 50-pound bags of garbage. So doing that for good part of time, you are like trying to breathe, trying to breathe. And it’s - I even told my staff. I’m all, hey, you know what? Let us stop, get somewhere cool, col- do-, cool down a little bit. Then we’ll go back out there, ‘cause I do not want nobody fainting or passing out.”*

*“When we have the mask on, and then we have, like, we have those backpack vacuum cleaners, and when the house is really hot, because a lot of the houses do not have- do not turn on their ACs on when we are cleaning. So like, just the breathing, trying to breathe with the mask on, working.”*

Some participants reported slowing down to address respiratory symptoms at work:

*“I slow down. I slow down. I have to slow down and catch my breath, then keep going. That’s all I can do.”*

Seven of the 11 participants reported concerns or issues with COVID-19. One participant described the experience of being on the frontlines early-on in the pandemic when little was known about the severity of COVID-19:

*“We risked our lives at the beginning, because we did not know, I mean, how bad the COVID was going to react to us.”*

Eight of the 11 participants were unaware of asthma as a risk from cleaning and disinfection, while others had considered the risks:

*“What’s the possible damages of bringing these chemicals, even though it says that it’s not super harmful to you, but after 2 years of spraying and spraying and spraying, what could be the damages? What are the possibilities of 5 years down the road from here? My lungs are, like, all filled with whatever the spraying we have been doing- is in my lungs now.”*

## Discussion

### Key findings

The prevalence of self-reported physician diagnosed asthma (14%) is higher in this community than in the general population (8.4%), although not statistically significant, potentially due to our small sample size (*n* = 59) or bias due to who was willing to take the survey. Self-reported physician diagnosed asthma was significantly (*p* < 0.05) associated with nine out of 17 respiratory symptoms, including missed days from work due to asthma in the last 12 months: Seventy-five percent (3/4) of participants with self-reported physician diagnosed asthma reported missed days, with only 5% (2/43) without self-reported physician diagnosed asthma reporting missed days. Our findings indicate that more research is needed to characterize asthmatic employees’ access to care and barriers to asthma management to increase their work stability, especially in populations composed of high proportions of immigrants and/or from racial or ethnic groups that are underserved. This is likely especially important when cleaning protocols include increased frequency of products or change in application types that include production of fogs or fine mists that may exacerbate asthma symptoms.

While reported change in cleaning protocol (yes/no), increased cleaning frequency, and change in type of cleaning product were not significantly associated with any respiratory symptoms, reported change in application type (e.g., fog, spray, wipe) was significantly associated with awakening during the night by attack/episode of cough. Interviews provided additional insight, with participants describing issues with the fogging/mechanical spraying devices that emitted a fine mist, causing both respiratory and skin issues for some. While this was implemented in response to COVID-19 to address fomite transmission, the risks of COVID-19 transmission via fomites are low (<1/10,000 per fomite touch). This elucidates an important risk–risk tradeoff: Are these misting devices and associated health issues for employees worth the relatively low COVID-19 risk reduction? These tradeoffs should be considered when implementing future cleaning and disinfection protocol changes in response to respiratory viral disease outbreaks.

Despite lack of significant relationships between reported increased cleaning frequency and respiratory symptoms, increased cleaning frequency was the most reported change in cleaning and disinfection protocols in both the survey (92%, 54/59) and in interviews (82%, 9/11). There is evidence from other studies that cleaning frequency and respiratory outcomes and dermatitis likely have a dose–response relationship (18) (i.e., increased frequency relating to increased risk), even for products that may pose less risk overall (e.g., “environmentally preferable” products relative to “traditional” products) (19). Although we do not demonstrate significant associations between respiratory symptoms and increased cleaning frequency, there is a theoretical reason to anticipate increased risk for workers with increased cleaning frequency. If the risks from fomites for a specific pathogen of concern are low, e.g., SARS-CoV-should be considered. An alternative approach to increased frequency is more focused cleaning efforts, such as the use of Targeted Hygiene (20), or focused cleaning on specific surfaces or moments (e.g., after a sick individual has occupied a given space), to optimize the benefits of cleaning and disinfection while minimizing exposure. However, more research is needed to evaluate how different overall cleaning and disinfection protocol approaches could reduce the burden of asthma on occupational groups that engage in cleaning and disinfection on a regular basis.

### Generalizability

To our knowledge, this is the first study to focus on the respiratory health of those in the cleaning industry as it relates to changes in cleaning and disinfection protocols during the COVID-19 pandemic. In a review of impacts of COVID-19 on environmental services personnel, findings included increased COVID-19 risks among environmental services personnel relative to other healthcare workers (21) and increased stress and anxiety (21). A participant in our study described the beginning of the pandemic as stressful, and noted a lack of messaging regarding appreciation for cleaning services personnel on the frontlines relative to other essential workers:

*“Like, for example, you know everybody, you know, during this whole pandemic, ‘Oh, thank you. Healthcare workers, thank you. Firemen, police officers. Thank you, mailman.’ But there wasn’t really a lot of that.”*

Other studies on those in the cleaning industry during COVID-19 indicated an increased need for PPE supplies and training to lower COVID-19 risks (22). Our study indicates that there are potential barriers to PPE use, including difficulty breathing with masks, especially in hot environments (e.g., cleaning homes in Arizona without air conditioning) and when conducting physically intensive tasks (i.e., lifting 20–50 lb. bags of garbage). Masks are often encouraged for asthmatic individuals during cleaning practices to protect them from inhalation of dust or other triggers (23). More research is needed to further elucidate the specific types of masks being used and barriers to proper use of PPE to increase successful implementation of COVID-19 interventions or interventions for asthmatic individuals to lower occupational risks for those in the cleaning industry.

### Limitations

While the sample sizes for both the survey (*n* = 59) and interviews (*n* = 11) were relatively small, little literature is available on the respiratory and occupational health of those in this industry, and no literature, to our knowledge, is available regarding their respiratory health during COVID-19, despite intensified cleaning and disinfection protocols and pre-pandemic evidence of respiratory risks for this occupational group. This is in part due to challenges in reaching workers from industries with generally higher proportions of racial and/or ethnic minority and/or immigrant community members. Sharing experiences and concerns regarding respiratory symptoms at work comes at a risk to the participants, including fears regarding job security, especially in Arizona. These types of studies also pose risks to employers, through whom recruitment is often the most successful or reliable (e.g., issues with reliability of data collected through surveys via social media or other online means). Our study reflects a new, yet successful, partnership with multiple businesses who expressed interest in bettering the occupational health of their employees. It is uncertain, however, whether the demographics of our participants represent those in other geographical areas or even in other cleaning occupations. Therefore, more research is needed to evaluate the effects of cleaning/disinfection protocol changes on the cleaning industry at large.

Due to potential concerns from participants about their privacy and job security, it is possible that our study underrepresents the severity of issues related to respiratory symptoms from cleaning and disinfection. For example, 22% (13/59) of survey participants preferred not to report whether the frequency of their respiratory symptoms at work had changed during COVID-19. While we do not know the reasons these participants chose not to respond, privacy and job security could be hypothesized. Conversely, it is also possible that our study overrepresents the severity of these issues, with those agreeing to participate reflecting individuals with the most extreme symptoms and a strong motivation to share their concerns. For example, a larger proportion of interview participants had self-reported physician diagnosed asthma (36%) than those who participated in the survey (14%) (Table 1), potentially indicating that those with asthma were more eager to share their experiences related to respiratory issues and cleaning/disinfection protocols at work than other employees. Furthermore, the proportion of participants in the survey who had asthma is higher than that found in the general population. While this may indicate an association between cleaning and disinfection exposures and asthma, it should be noted that prior work-related respiratory issues could be leading to exacerbated respiratory symptoms as opposed to exposures related to cleaning and disinfection protocol changes. COVID-19 infection, for example, could drive changes in respiratory symptoms as opposed to cleaning and disinfection protocols that were changed. Continued collaboration and building of trust with this community, both employers and employees, is needed to further explore potential confounders and sources of selection bias (e.g., those with respiratory symptoms more likely to participate) to accurately characterize the true burden of respiratory disease, especially as it relates specifically to occupational exposures.

Due to potential technology accessibility challenges, employers encouraged the use of hard-copy surveys in this study. These surveys were anonymous, apart from a provided email address to receive compensation. This introduced some uncertainty regarding whether one individual filled out only one survey and whether individuals were able to read and comprehend all survey questions. However, our approach was the most feasible way to recruit from this population during the COVID-19 pandemic, due to restrictions on meeting in person. Prior to COVID-19, a more reliable strategy would include in-person verbal surveys to ensure participants’ comprehension of the questions and to ensure there was only one completed survey per individual. This study highlights the challenges of conducting needed COVID-19-related research with underserved populations.

## Conclusion

This study provides preliminary evidence that the burden of asthma among those in maid, janitor, or custodial positions may be higher than the general population, and that changes in cleaning and disinfection protocols during COVID-19 (namely, the use of fogging/mechanical spraying devices) may have had negative impacts on the health of workers in the cleaning industry with little benefit to reducing COVID-19 risks. However, important confounders require further investigation, such as the effect of COVID-19 infection on the respiratory health of those in the cleaning industry. Other key findings included hardships regarding challenges with PPE use. Further research is needed to evaluate the generalizability of our findings across larger geographical areas beyond Arizona and to develop guidance for employers and employees on how to protect and promote the respiratory health of those in the cleaning industry.

## Data availability statement

The raw anonymized data supporting the conclusions of this article will be made available by the authors, without undue reservation.

## Ethics statement

The studies involving humans were approved by the University of Arizona Institutional Review Board. The studies were conducted in accordance with the local legislation and institutional requirements. The ethics committee/institutional review board waived the requirement of written informed consent for participation from the participants or the participants’ legal guardians/next of kin because this was done to protect participant identity. Participants were provided a consent document but a signature was not collected.

## Author contributions

AW conceived the project, led the research and manuscript writing, conducted the English interviews and quantitative analysis of survey data, contributed to analysis of English interview transcripts, and obtained funding to support the project. YJ contributed to English interview transcription and analysis. SM and IK contributed to Spanish interview transcription, analysis, and translation. JE contributed to English interview transcription. CR conducted Spanish interviews. VV contributed to the quantitative analysis of survey data. CR and SS contributed to Spanish translations of survey materials and interview questions. LG provided senior mentor oversight and contributed to the participant recruitment plan. PB provided senior mentor oversight, contributed to the participant recruitment plant, and contributed to translation of materials into Spanish. All authors contributed to the article and approved the submitted version.

## Funding

This research was funded by a small research project grant through the National Heart, Lung, and Blood Institute-Funded Program: Programs to Increase Diversity Among Individuals Engaged in Health Related Research (PRIDE) Advanced Respiratory Research for Equity (AIRE) program (Grant No. NHLBI 5R25HL126140-06), of which AW was a participant. AW was supported through a University of Arizona Health Sciences Career Development Award. AW, SS, and PB were supported by the Southwest Environmental Health Sciences Center (NIEHS P30 ES006694). The content was solely the responsibility of the authors and does not necessarily represent the official views of the National Institutes of Health.

## Conflict of interest

The authors declare that the research was conducted in the absence of any commercial or financial relationships that could be construed as a potential conflict of interest.

## Publisher’s note

All claims expressed in this article are solely those of the authors and do not necessarily represent those of their affiliated organizations, or those of the publisher, the editors and the reviewers. Any product that may be evaluated in this article, or claim that may be made by its manufacturer, is not guaranteed or endorsed by the publisher.

## Supplementary material

The Supplementary material for this article can be found online at: https://www.frontiersin.org/articles/10.3389/fpubh.2023.1181047/full#supplementary-material

## References

[1] Rosenman K Reilly MJ Pechter E Fitzsimmons K Flattery J Weinberg J. Cleaning products and work-related asthma, 10 year update. J Occup Environ Med. (2020) 62:130–7. doi: 10.1097/JOM.0000000000001771 31895737 PMC7839059

[2] Romero Starke K Friedrich S Schubert M Kämpf D Girbig M Pretzsch A. Are healthcare workers at an increased risk for obstructive respiratory diseases due to cleaning and disinfection agents? A systematic review and meta-analysis. Int J Environ Res Public Health. (2021) 18:5159. doi: 10.3390/ijerph18105159, PMID: 34068014 PMC8152277

[3] Dumas O Wiley AS Quinot C Varraso R Zock JP Henneberger PK. Occupational exposure to disinfectants and asthma control in US nurses. Eur Respir J. (2017) 50:1700237. doi: 10.1183/13993003.00237-2017, PMID: 28982772 PMC5702691

[4] Dumas O Gaskins AJ Boggs KM Henn SA le Moual N Varraso R. Occupational use of high-level disinfectants and asthma incidence in early- to mid-career female nurses: a prospective cohort study. Occup Environ Med. (2021) 78:244–7. doi: 10.1136/oemed-2020-106793, PMID: 33452037 PMC7985390

[5] Gonzalez M Jégu J Kopferschmitt MC Donnay C Hedelin G Matzinger F. Asthma among workers in healthcare settings: role of disinfection with quaternary ammonium compounds. Clin Exp Allergy. (2014) 44:393–406. doi: 10.1111/cea.12215, PMID: 24128009

[6] Zock JP Vizcaya D Le Mouai N. Update on asthma and cleaners. Curr Opin Allergy Clin Immunol. (2010) 10:114–20. doi: 10.1097/ACI.0b013e32833733fe 20093933 PMC3125175

[7] Medina-Ramón M Zock JP Kogevinas M Sunyer J Antó JM. Asthma symptoms in women employed in domestic cleaning: a community based study. Thorax. (2003) 58:950–4. doi: 10.1136/thorax.58.11.950 14586047 PMC1746523

[8] Carder M Seed MJ Money A Agius RM van Tongeren M. Occupational and work-related respiratory disease attributed to cleaning products. Occup Environ Med. (2019) 76:530–6. doi: 10.1136/oemed-2018-105646 31167951

[9] Gharpure R Hunter CM Schnall AH Barrett CE Kirby AE Kunz J. Knowledge and practices regarding safe household cleaning and disinfection for COVID-19 prevention - United States, May 2020. MMWR Morb Mortal Wkly Rep. (2020) 69:705–9. doi: 10.15585/mmwr.mm6923e2 32525852 PMC7315790

[10] Miller SL Nazaroff WW Jimenez JL Boerstra A Buonanno G Dancer SJ. Transmission of SARS-CoV-2 by inhalation of respiratory aerosol in the Skagit Valley chorale superspreading event. Indoor Air. (2020) 31:314–23. doi: 10.1111/ina.12751 32979298 PMC7537089

[11] Wilson AM Sleeth DK Schaefer C Jones RM. Transmission of respiratory viral diseases to health care workers: COVID-19 as an example. Annu Rev Public Health. (2022) 43:311–30. doi: 10.1146/annurev-publhealth-052120-110009, PMID: 34995130 PMC12699781

[12] Jones RM. Relative contributions of transmission routes for COVID-19 among healthcare personnel providing patient care. J Occup Environ Hyg. (2020) 17:408–15. doi: 10.1080/15459624.2020.1784427, PMID: 32643585

[13] Wilson AM Weir MH Bloomfield SF Scott EA Reynolds KA. Modeling COVID-19 infection risks for a single hand-to-fomite scenario and potential risk reductions offered by surface disinfection. Am J Infect Control. (2021) 49:846–8. doi: 10.1016/j.ajic.2020.11.013 33207258 PMC7666808

[14] Pitol AK Julian TR. Community transmission of SARS-CoV-2 by surfaces: risks and risk reduction strategies. Environ Sci Technol Lett. (2021) 8:263–9. doi: 10.1021/acs.estlett.0c00966 37566313

[15] Harvey AP Fuhrmeister ER Cantrell ME Pitol AK Swarthout JM Powers JE. Longitudinal monitoring of SARS-CoV-2 RNA on high-touch surfaces in a community setting. Environ Sci Technol Lett. (2021) 8:168–75. doi: 10.1021/acs.estlett.0c00875, PMID: 34192125 PMC7927285

[16] Delclos GL Arif AA Carson A Lai D Lusk C Stock T. Validation of an asthma questionnaire for use in healthcare workers. Occup Environ Med. (2006) 63:173–9. doi: 10.1136/oem.2005.021634 16497858 PMC2078145

[17] National Center for Environmental Health. Most Recent National Asthma Data. (2022). Available at: https://www.cdc.gov/asthma/most_recent_national_asthma_data.htm (Accessed February 15, 2023).

[18] Arif AA Delclos GL. Association between cleaning-related chemicals and work-related asthma and asthma symptoms among healthcare professionals. Occup Environ Med. (2012) 69:35–40. doi: 10.1136/oem.2011.064865 21602538

[19] Garza JL Cavallari JM Wakai S Schenck P Simcox N Morse T. Traditional and environmentally preferable cleaning product exposure and health symptoms in custodians. Am J Ind Med. (2015) 58:988–95. doi: 10.1002/ajim.22484, PMID: 26040239 PMC4976595

[20] Bloomfield SF Ackerley LM. Developing resilience against the threat of infectious diseases and anti-microbial resistance: putting targeted hygiene into practice in home and everyday lives. Public Health Practi. (2023) 5:100362. doi: 10.1016/j.puhip.2023.100362 PMC9869640 36712152

[21] Ng QX Yau CE Yaow CYL Lim YL Xin X Thumboo J. Impact of COVID-19 on environmental services workers in healthcare settings: a scoping review. J Hosp Infect. (2022) 130:95–103. doi: 10.1016/j.jhin.2022.09.001, PMID: 36116538 PMC9474977

[22] Daba C Gebrehiwot M Asefa L Lemma H Atamo A Kebede E. Occupational safety of janitors in Ethiopian university during COVID-19 pandemic: results from observational study. Front Public Health. (2022) 10:895977. doi: 10.3389/fpubh.2022.895977, PMID: 35968437 PMC9374277

[23] Asthma and Allergy Foundation of America. Cleaning when you have asthma: the dirty truth. (2021). Available at: https://community.aafa.org/blog/cleaning-when-you-have-asthma-the-dirty-truth (Accessed February 15, 2023).

