# Supplementary material for: COVID-19 cleaning protocol changes, experiences, and respiratory symptom prevalence among cleaning services personnel
Source: Front Public Health. 2023 Sep 14;11:1181047. doi: 10.3389/fpubh.2023.1181047 (PMC10538963; doi:10.3389/fpubh.2023.1181047)
Supplement: Supplementary file 1 [file Table_1.docx]

**SUPPLEMENTAL MATERIAL**

**for**

**COVID-19 cleaning protocol changes, experiences, and respiratory symptom prevalence among cleaning services personnel**

**Table S1. Categorization of interview transcript data**

| Gender (open-ended) | |
| --- | --- |
| Race (open-ended) | |
| Ethnicity | Hispanic |
|  | Non-Hispanic |
| Age (continuous) | |
| Physician diagnosed asthma | Not diagnosed |
|  | Diagnosed as adult |
|  | Diagnosed as child |
|  | Implied they no longer had asthma |
| Type of environment (open-ended) |  |
| Type of cleaning at work | Handheld spray |
|  | Wipes |
|  | Fogging/Motorized sprayer |
|  | Other |
| Type of cleaning at home | Spray |
|  | Wipes |
|  | Other |
| Type of surfaces cleaned at work (open-ended) | |
| Type of surfaces cleaned at home (open-ended) | |
| Changes in cleaning/disinfection protocols at work due to COVID-19 | Change in cleaning frequency |
|  | PPE changes |
|  | Change in product |
|  | Change in application type |
|  | Change in type of surface |
|  | Heightened awareness |
|  | Other |
|  | None |
| Challenges/strategies related to PPE or respiratory symptoms at work | Difficulty breathing in mask due to heat |
|  | Use of medication |
|  | Needed skin protection |
|  | Slowing down if having respiratory symptoms |
|  | Other |
|  | None |
| Challenges/strategies related to PPE or respiratory symptoms at home | Difficulty breathing in masks due to heat |
|  | Reaction to pets |
|  | Use of medication |
|  | Slowing down if having respiratory symptoms |
|  | None |
| Concerns/issues with inhalation of cleaning/disinfection chemicals at work | Yes |
|  | No |
| Other symptoms or concerns from cleaning/disinfection at work | Skin |
|  | Nose irritation |
|  | Eye irritation |
|  | Cancer |
|  | Other |
|  | None |
| Concerns/issues with inhalation of cleaning/disinfection chemicals at home | Yes |
|  | No |
| Other symptoms or concerns from cleaning/disinfection at home | Skin |
|  | Nose irritation |
|  | Eye irritation |
|  | Cancer |
|  | None |
| Concerns/issues with COVID-19 | Yes |
|  | No |
| Risk-risk tradeoff thoughts related to cleaning and disinfection (increased respiratory risks for increased cleaning and disinfection, increased infection risks for decreased cleaning and disinfection) (open-ended) | |
| Awareness of asthma risk from cleaning and disinfection activity | Yes |
|  | No |
|  | Uncertain (not enough data to categorize) |
